# Characterization of ACE and ACE2 Expression within Different Organs of the NOD Mouse

**DOI:** 10.3390/ijms18030563

**Published:** 2017-03-05

**Authors:** Heleia Roca-Ho, Marta Riera, Vanesa Palau, Julio Pascual, Maria Jose Soler

**Affiliations:** 1 Institut Hospital del Mar d’Investigacions Mèdiques, 08003 Barcelona, Spain; heleia.roca@gmail.com (H.R.-H.); mriera1@imim.es (M.R.); vpalau@imim.es (V.P.); jpascualsantos@parcdesalutmar.cat (J.P.); 2 Nephrology Department—Hospital del Mar and Institut Hospital del Mar d’Investigacions Mèdiques—IMIM, 08003 Barcelona, Spain

**Keywords:** renin angiotensin system, angiotensin converting enzyme 2, diabetes

## Abstract

Renin angiotensin system (RAS) is known to play a key role in several diseases such as diabetes, and renal and cardiovascular pathologies. Its blockade has been demonstrated to delay chronic kidney disease progression and cardiovascular damage in diabetic patients. In this sense, since local RAS has been described, the aim of this study is to characterize angiotensin converting enzyme (ACE) and ACE2 activities, as well as protein expression, in several tissues of the non-obese diabetic (NOD) mice model. After 21 or 40 days of diabetes onset, mouse serums and tissues were analyzed for ACE and ACE2 enzyme activities and protein expression. ACE and ACE2 enzyme activities were detected in different tissues. Their expressions vary depending on the studied tissue. Thus, whereas ACE activity was highly expressed in lungs, ACE2 activity was highly expressed in pancreas among the studied tissues. Interestingly, we also observed that diabetes up-regulates ACE mainly in serum, lung, heart, and liver, and ACE2 mainly in serum, liver, and pancreas. In conclusion, we found a marked serum and pulmonary alteration in ACE activity of diabetic mice, suggesting a common regulation. The increase of ACE2 activity within the circulation in diabetic mice may be ascribed to a compensatory mechanism of RAS.

## 1. Introduction

Angiotensin converting enzyme (ACE)2 is an enzymatically active ACE homologue, which shares 42% of its amino acidic sequence identity in its catalytic domain; however, ACE and ACE2 show several differences. Whereas ACE is a dipeptidylcarboxypeptidase, presenting both N- and C-terminus catalytic domains with two zinc-binding motifs (HEXXH, where X is any amino acid); ACE2 is a monocarboxypeptidase with only one zinc-binding motif at its N-terminal domain [1,2,3]. Through this catalytic domain, ACE2 hydrolyzes AngII (angiotensin II) to generate Ang1–7, a peptide that binds to the MAS receptor (Ang1-7 receptor) and activates vasodilation, anti-fibrosis, anti-proliferation, and anti-inflammatory effects as well as counterbalances the ACE-AngII-ATR1 axis actions [4,5,6].

Renin angiotensin system (RAS) was classically described as a regulation system responsible for blood pressure regulation, electrolyte, and fluid homeostasis, exerting most of its actions through kidneys [7]. Later on, its elements were also found in extrarenal tissues, indicating the presence of a local paracrine system that coexists with the circulating RAS [8]. Thus, ACE expression was mainly found in the surface of endothelial cells of lungs, renal brush border membranes, intestines, choroid plexus, placenta [9,10,11], and, to a lesser extent, in cardiac, hepatic, pancreatic, and adrenal tissues [12]. In contrast, ACE2 protein expression was initially reported to be more tissue restricted and higher than ACE, being described preferentially in kidneys (renal tubules and glomeruli), heart, and testis [13]. Subsequently, ACE2 expression was also found widespread in many other organs such as lungs, pancreas [14,15], bladder, stomach, ileum, adipocytes, and liver [16,17].

RAS activation is known to play a key role in several diseases, namely diabetes, and renal and cardiovascular pathologies [18,19]. ACE2 and ACE enzymes have found to be altered in the kidney from different diabetic experimental models, the *db*/*db* (type 2) and streptozotozin (STZ) (type 1) diabetic mice [20,21,22]. However, ACE2 and ACE enzymes have not been extensively studied in other tissues from diabetic mice. The non-obese diabetic (NOD) mice is a strain that spontaneously develops autoimmune diabetes that mimics type 1 diabetes in humans [23,24]. We hypothesized that ACE2 and ACE enzymes are present in different tissues other than kidney and differentially expressed. In addition, the administration of insulin can alter ACE2 and ACE expression within these tissues from NOD mice. For this purpose, we studied both ACE and ACE2 activities as well as their protein expression in several tissues from NOD diabetic mice. We also analyzed the effect of diabetes on ACE2 and ACE modifications in tissues from NOD diabetic mice as compared to non-obese resistant (NOR) mice. In addition, we assessed the effect of insulin administration on ACE2 and ACE expression in the studied tissues.

## 2. Results

### 2.1. Establishing Buffer Assay and Optimal Amounts of Protein for ACE and ACE2 Activities Measurement in Several Tissues

**Figure 1:**
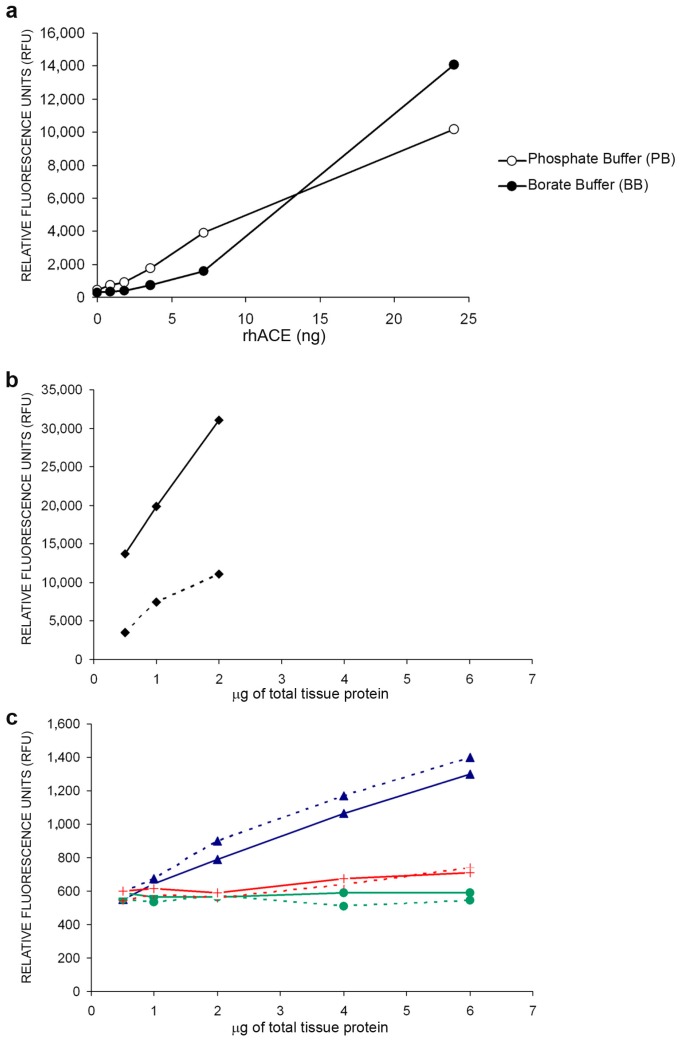
Implementation of angiotensin converting enzyme (ACE) activity assays with different buffers and in different tissues. (**a**) ACE activity with borate (BB) and phosphate buffer (PB) after increasing amounts (ng) of human recombinant ACE (hrACE). ACE activity at lower concentrations of hrACE was only detected by the use of BB; (**b**) ACE activity in lung measured with BB (black rhombus), and (**c**) ACE activity in heart (blue triangle), pancreas (green circle), and liver (red cross) from control (dashed lines) and diabetic (continuous lines) mice. Increasing amounts of micrograms of total protein extracts were tested.

ACE enzymatic determination technique was set up using two buffers. For this study, human recombinant ACE (hrACE) was used. Phosphate Buffer (PB) and Borate Buffer (BB) were incubated with increasing amounts of hrACE (Figure 1a). We observed that ACE activity at lower hrACE amounts was only detected by the use of BB. In addition, the hrACE activity measurement was found to be linear with BB as compared to PB. PB needed higher amounts of hrACE for its detection, suggesting a lower sensitivity when this buffer was used.

**Figure 2:**
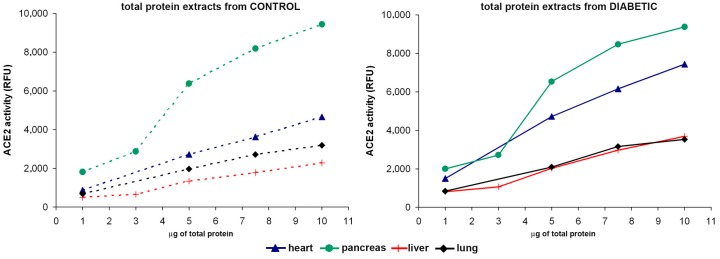
ACE2 activity assays in different tissues and with increasing protein amounts. ACE2 activity was measured in lung (black rhombus), heart (blue triangle), pancreas (green circle), and liver (red cross) from control animals (CONT) (**left panel**) and diabetic animals (DB) (**right panel**) mice. Increasing amounts of micrograms of total protein extracts from each studied tissue were tested (1, 3, 5, 7.5, and 10 μg of protein).

After the above-mentioned experiments, we decided to use BB for our ACE activity assays. The next step was to test the optimal amounts of tissue protein for ACE activity measurements (Figure 1b,c). Increasing amounts of protein for each tissue of both control (CONT, dashed lines) and diabetic (DB, continuous lines) mice were used. Pulmonary tissue presented a linear enzymatic activity in both CONT and DB mice due to its high enzymatic activity (Figure 2b). In contrast, no linearity in increasing amounts of heart (triangle), pancreas (circle), and liver (cross) samples due to the low ACE activity detection was observed (Figure 1c). Furthermore, it is worth noting that ACE activity in DB was higher as compared to CONT, and lung presented the highest levels of ACE activity.

ACE2 enzymatic activity was already previously established and validated in our laboratory by using a specific ACE2 quenched fluorogenic substrate [25,26,27]. The optimal amount of each studied tissue was determined by reactions with increasing amounts of protein in pulmonary, cardiac, hepatic, and pancreatic tissues of both CONT and DB samples (Figure 2). Higher ACE2 activity was observed in lung, heart, liver, and pancreas from CONT mice, with increased amounts of protein. Interestingly, increased levels of enzymatic activities were found in tissue samples from DB mice as compared to controls. Among all studied tissues, pancreas presented higher levels of ACE2 activity followed by heart, lung, and liver.

### 2.2. ACE Activity in Serum, Lung, Heart, Liver, and Pancreas from Diabetic, Insulin-Treated, and Control Mice

**Figure 3:**
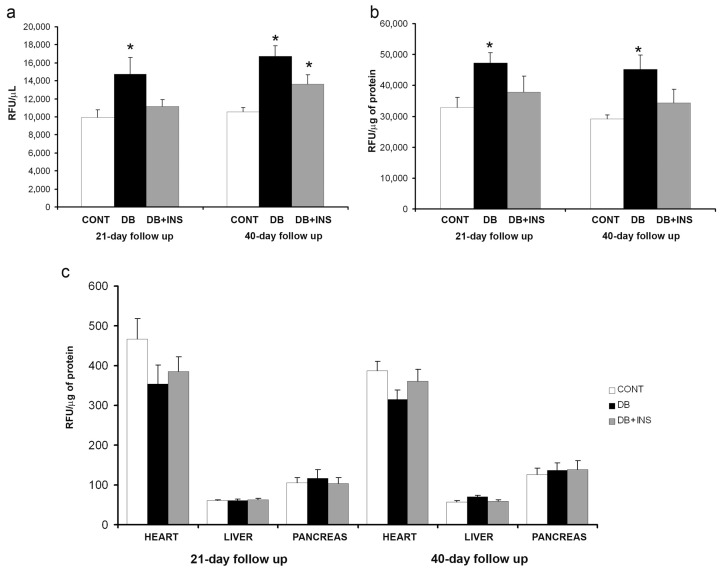
ACE activity in serum and different tissues. (**a**) ACE activity in serum samples from CONT (open bars), DB (closed bars), and insulin-treated diabetic (DB + INS, grey bars) mice at 21 and 40 days after diabetes onset; (**b**) ACE activity in lungs from CONT, DB, and DB + INS mice; (**c**) ACE activity in heart, liver, and pancreas from CONT, DB, and DB + INS. * *p* ≤ 0.05 CONT vs DB.

In serum samples, ACE activity was significantly increased in DB at early and late stages of diabetes as compared to CONT mice (*p* = 0.04 in 21-day follow up and *p* = 0.0003 in 40-day follow up). Insulin administration slightly decreased circulating ACE activity in DB mice at both early and late stages (*p* = NS, not significant) (Figure 3a). ACE activity was significantly increased in lungs from DB mice at early and late stages of diabetes as compared to CONT mice (*p* = 0.045 and *p* = 0.026, respectively). Insulin administration did not modify ACE activity in lungs from DB as compared to CONT mice (*p* = NS) (Figure 3b). Regarding heart samples, no statistically significant differences were observed in ACE activity in DB as compared to their respective CONT mice *(p* = NS). Furthermore, no significant effect was observed in cardiac tissue after insulin treatment in DB as compared to CONT mice (*p* = NS) (Figure 3c). ACE activity was also tested in liver. As observed in heart, no differences in ACE activity levels between DB and CONT mice were found in liver tissue (*p* = NS). No changes were observed after insulin administration (*p* = NS) (Figure 3c). No significant differences in ACE activity levels were found in pancreas from DB and CONT mice (*p* = NS) (Figure 3c).

### 2.3. ACE2 Activity in Serum, Lung, Heart, Liver, and Pancreas from Diabetic, Insulin-Treated, and Control Mice

**Figure 4:**
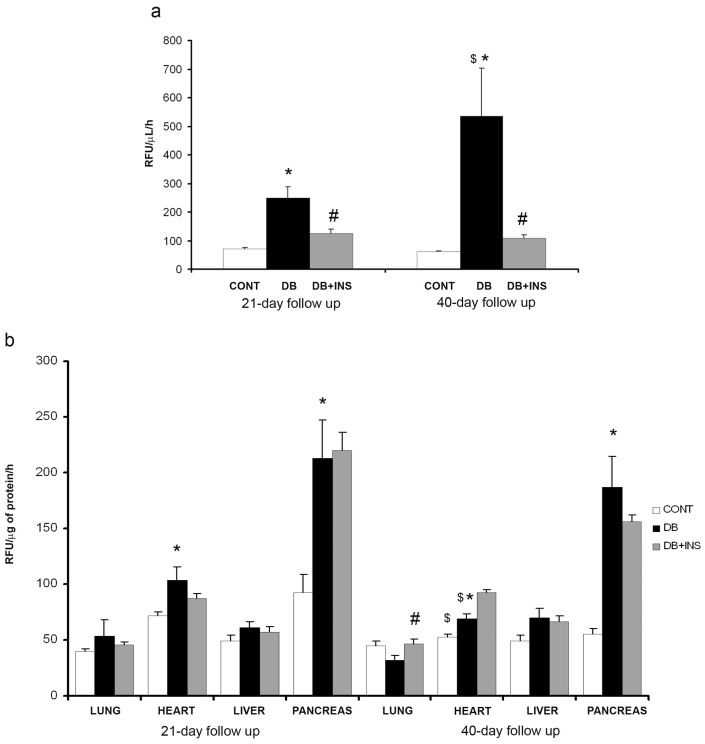
ACE2 activity in serum and different tissues. (**a**) ACE2 activity in serum samples from CONT (open bars), DB (closed bars), and DB + INS (grey bars) mice at 21 and 40 days after diabetes onset; (**b**) ACE2 activity in lung, heart, liver, and pancreas from CONT, DB, and DB + INS. * *p* ≤ 0.05 CONT vs. DB; ^#^ *p* ≤ 0.05 DB vs. DB + INS; ^$^ *p* ≤ 0.05 21 vs. 40 days of follow up.

In serum samples, there was a significant increase of ACE2 activity in DB mice at early and late stages of diabetes as compared to CONT (*p* = 0.0003 and *p* = 0.0003, respectively) and insulin administration significantly decreased ACE2 activity in DB mice at early and late stages (*p* = 0.001 and *p* = 0.001, respectively) (Figure 4a). In lung homogenates, no changes were observed between DB and CONT mice (*p* = NS). Interestingly, insulin administration significantly increased ACE2 activity in DB mice at late stage as compared to DB mice (*p* = 0.05) (Figure 4b). At cardiac level, there was a significant increase of ACE2 activity in DB mice in both early and late stages of DB as compared to CONT mice (*p* = 0.011 and *p* = 0.029, respectively), however, insulin administration did not modify this pattern (*p* = NS) (Figure 4b). In liver samples, no changes were observed between the studied groups (Figure 4b). Pancreatic ACE2 activity was increased in DB mice as compared to CONT mice (*p* = 0.014 in 21-day follow up and *p* = 0.0003 in 40-day follow up of study). In addition, insulin administration did not change ACE2 activity in pancreas from DB mice (*p* = NS) (Figure 4b).

### 2.4. ACE2/ACE Activity Ratio in Serum, Lung, Heart, Liver, and Pancreas from Diabetic, Insulin-Treated, and Control Mice

**Figure 5:**
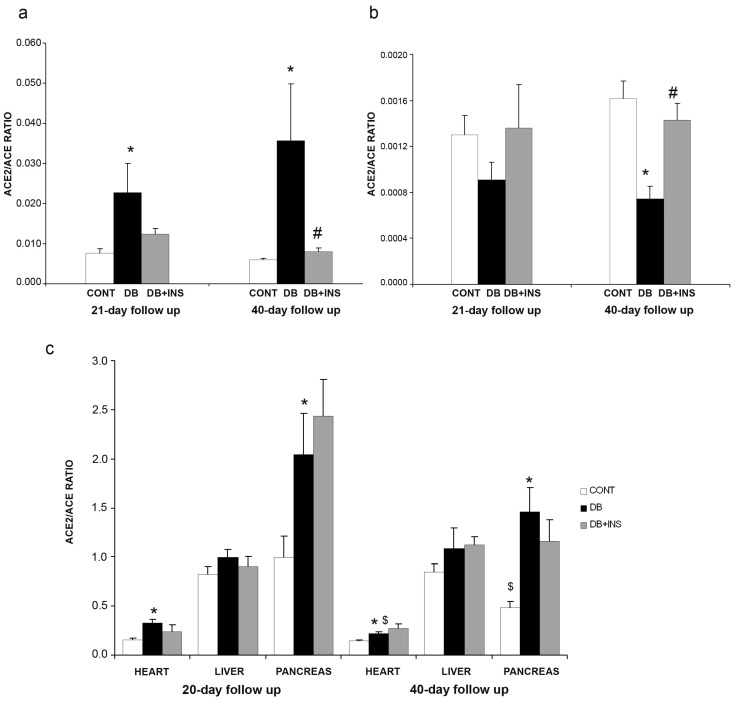
ACE2/ACE activity ratio in serum and different tissues. (**a**) ACE2/ACE activities ratio in serum samples from CONT (open bars), DB (closed bars), and DB + INS (grey bars) mice at 21 and 40 days after diabetes onset; (**b**) ACE2/ACE activity ratio in lungs from CONT, DB, and DB + INS mice; (**c**) ACE2/ACE activity ratio in heart, liver, and pancreas from CONT, DB, and DB + INS. * *p* ≤ 0.05 CONT vs. DB; ^#^ *p* ≤ 0.05 DB vs. DB+INS; ^$^ *p* ≤ 0.05 21 vs. 40 days of follow up.

After ACE and ACE2 activities were measured, ACE2/ACE activity ratios were calculated for each studied tissue to infer the status of RAS in different tissues as an ACE-ACE2 balance. In serum samples, ACE2/ACE activity ratio in DB mice at early and late stages was higher as compared to CONT (*p* = 0.011 and *p* = 0.0003, respectively). Insulin administration significantly decreased circulating ACE2/ACE activity ratio in DB mice (*p* = 0.001) (Figure 5a). In lung, there were no differences in ACE2/ACE activity ratio between the DB and CONT groups at early stage. Interestingly, ACE2/ACE activity ratio was significantly decreased in DB at late stage as compared to CONT mice (*p* = 0.001). Insulin administration significantly increased ACE2/ACE activity ratio in DB mice (*p* = 0.014) (Figure 5b). At cardiac level, ACE2/ACE activity ratio was significantly increased in DB mice in both early and late stages as compared to CONT mice (*p* = 0.0003 and *p* = 0.008, respectively), but insulin administration had no effect on ACE2/ACE activity in DB (Figure 5c). In pancreas, ACE2/ACE activity ratio was significantly increased in DB mice in both early and late stages as compared to CONT mice (*p* = 0.05 and *p* = 0.001, respectively), but insulin administration had no effect on ACE2/ACE activity in DB (Figure 5, panel c). In liver, there were no differences in ACE2/ACE activity ratio in DB as compared to their respective CONT (*p* = NS). Insulin administration did not modify ACE2/ACE activity in liver (Figure 5c).

### 2.5. ACE and ACE2 Protein Expression in Heart, Lung, Liver, and Pancreas from Diabetic, Insulin-Treated, and Control mice

**Figure 6:**
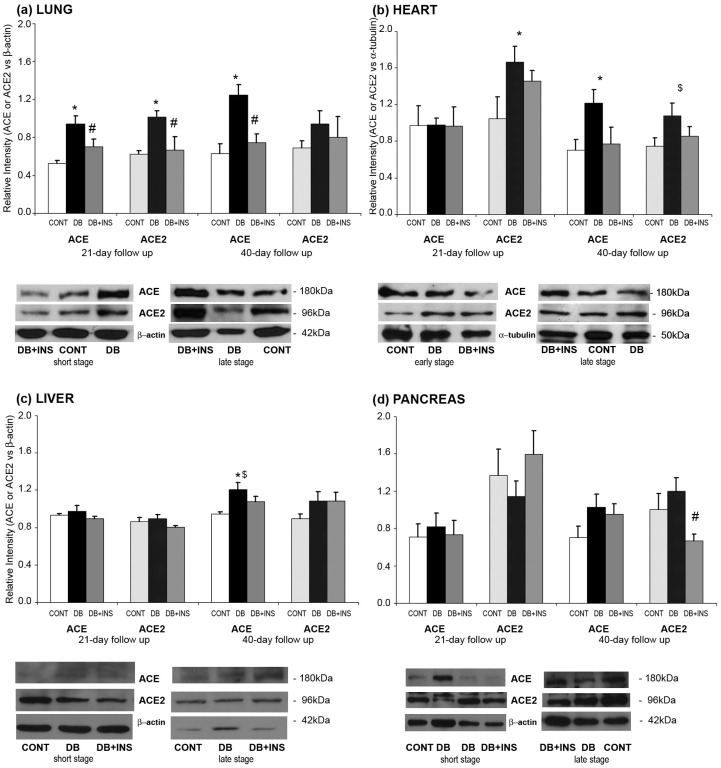
ACE and ACE2 protein expression in different tissues. Upper panel: normalized ACE and ACE2 protein expression in lung (**a**); heart (**b**); liver (**c**); and pancreas (**d**) homogenates from CONT (open bars), DB (closed bars), and DB + INS (grey bars) mice at 21 and 40 days after diabetes onset. Lower panel: representative images depicting bands corresponding to ACE and ACE2 and protein controls. * *p* ≤ 0.05 CONT vs. DB; ^#^ *p* ≤ 0.05 DB vs. DB + INS; ^$^ *p* ≤ 0.05 21 vs. 40 days of follow up.

To assess ACE protein expression, immunoblotting techniques were performed. In lungs, ACE protein expression was significantly increased in DB mice at early and late stages as compared to CONT (*p* = 0.008 and *p* = 0.012, respectively). In addition, insulin administration significantly decreased ACE protein expression in DB mice (*p* = 0.05 and *p* = 0.024, respectively) (Figure 6a). In heart, ACE protein expression was significantly increased in DB mice at late stage as compared to CONT (*p* = 0.03) (Figure 6b). Insulin administration did not modify ACE protein expression. In liver and pancreas, there were no differences between DB and CONT mice (Figure 6c).

In lungs, ACE2 protein expression was significantly increased in DB mice at early stage as compared to CONT mice (*p* = 0.008). In addition, insulin administration significantly decreased ACE2 protein expression in DB mice (*p* = 0.05) (Figure 6a). In heart, ACE2 protein expression was significantly increased in DB mice at early stage as compared to CONT mice (*p* = 0.022 and *p* = 0.035, respectively). Insulin administration did not modify ACE2 protein expression in DB mice (*p* = NS) (Figure 6b). In liver, ACE2 protein expression was significantly increased in DB mice at late stage as compared to CONT mice (*p* = 0.008) and insulin administration did not modify ACE2 protein expression (Figure 6c). In pancreas, there were no changes observed when ACE2 protein expression was studied (Figure 6d).

### 2.6. Correlation between Blood Glucose Levels and ACE2/ACE Activity Ratio among Different Tissues

Mean blood glucose levels in control animals were 160.25 ± 9.47 mg/dL at the end of the study. In diabetic animals, blood glucose levels were significantly increased to 550 ± 33.08 mg/dL in animals followed for 21 days and 555 ± 3.47 mg/dL in the animals followed for 40 days. Insulin pellets significantly reduced blood glucose levels at 110.65 ± 11 mg/dL and 97.67 ±9.48 mg/dL for 21 and 40 days of diabetes, respectively.

Blood glucose levels positively correlated with ACE2/ACE ratio in serum (*r* = 0.455, *p* = 0.003) and negatively correlated with ACE2/ACE ratio in lung (*r* = −0.546, *p* = 0.0005).

## 3. Discussion

Several works have been focused on the study of ACE and ACE2 enzymes within the diabetic kidney [21,28]. ACE2 has been shown to be increased in the kidney from different models of diabetic nephropathy, the STZ-diabetic model and the non-obese diabetic mice (NOD), among others [20,25]. However, in other tissues from diabetic animals, such as liver and heart, ACE and ACE2 enzymes have not been widely studied. In the present study, we demonstrated that ACE and ACE2 activities are present in different tissues. Interestingly their expression is different depending on the tissue: ACE is highly expressed in lung, whereas ACE2 is highly expressed in pancreas among the studied tissues. We also observed that diabetes up-regulated ACE and ACE2 activity and protein expression in the majority of the studied tissues.

A large number of ACE activity detection techniques have been described, such as radioassays, high-performance liquid chromatography, and colorimetric-based assays [29,30,31]. Of note that, ACE activity measurement based on fluorimetric quantification is the most widely used technique because of its sensitivity, simplicity, speed, and high reproducibility [32]. In 1971, Cushman and Cheung developed an ACE activity assay using synthetic ACE-specific substrates, including one of the substrates most commonly cited in literature, hippuryl-l-histidyl-l-leucine (HHL) [33]. As an indirect determination, this technique is based on the hydrolysis of HHL and the measurement of fluorescence through the *o*-phthalaldialdehyde adducts formation with HHL [34]. In this work, ACE activity was indirectly measured using HHL as the substrate for ACE, as previously described. Previous studies have shown that ACE activity from the same samples differs depending on the homogenization buffer used [32,35]. We now show that borate buffer is the most suitable to detect lower levels of ACE activity, showing better linearity, conferring less variability, and offering a more reliable assay.

Our results showed that ACE activity in lungs was higher as compared to other studied tissues. Moreover, a significant increase was observed in serum and lung from diabetic mice at early and late stages as compared to control mice. The same pattern was observed when ACE protein expression was studied. These observations are consistent with previous studies from Huang and co-authors showing increased ACE activity in plasma and mRNA levels in lung from STZ-induced diabetic C57BL/6 mice [36]. Thus, there is a coupled (serum and pulmonary) alteration in ACE activity, suggesting that pulmonary and circulating RAS may exert a common regulation.

Since RAS has a key role in cardiovascular diseases, recent studies have associated RAS with liver fibrosis, cirrhosis, and portal pressure regulation [37]. Interestingly, pancreas is found to express local RAS. The role of RAS components in diabetes have been previously studied [15,25,38]. In addition, ACE was also increased in liver from DB mice at late stage of follow-up. We found that ACE activity was decreased in heart from DB mice, but only at early stage. However, in previous studies by Colucci and co-authors, there were no differences between control and diabetic mice [12]. In all studied tissues, the insulin-treated group presented no significant differences compared to the non-treated diabetic group.

ACE2 enzyme, a novel ACE homologue, was discovered in the last decade [1,2]. For years, the detection of endogenous ACE2 activity in mouse tissues was difficult and ACE2 activity assay is a relatively recent reliable technique [39,40]. ACE2 activity was performed in serum, lung, heart, liver, pancreas, and kidney tissues by measuring the hydrolysis of Mca-APK(Dnp), a quenched-fluorescent specific ACE2-substrate.

Our findings showed that ACE2 activity levels were higher in pancreas as compared to other studied tissues. Interestingly, significant increases in serum, pancreas, and heart in ACE2 activity from NOD diabetic mice as compared to NOR mice at 21 and at 40 days after the onset of diabetes were observed. In addition, ACE2 activity was also increased in liver at 40 days of diabetes in NOD mice. With regard to protein expression, ACE2 expression levels were increased in lungs and heart at early stage of diabetes and in pancreas at late stage of diabetes. Previous studies postulated that the differences observed in ACE2 activities are related to ADAM17 sheddase activity [41]. However, no differences were found when studying ACE2 and ADAM17 activities and gene expression in pancreas islet from *db*/*db* mice as compared to the respective *db*/*m* controls [42].

It is of note that insulin administration mainly restored ACE and ACE2, and ACE2/ACE ratio activities in serum samples at longer time of follow-up. However, these results were not consistent in other tissues. These findings may be ascribed to the direct correlation observed between glucose levels and ACE2/ACE ratio. Thus, the results observed may indicate in part a protective effect of insulin on normalizing circulating RAS activities.

In conclusion, we assessed ACE and ACE2 activities in different tissues of NOD mice and demonstrated that ACE activity is highly detected in lungs, whereas ACE2 activity is highly detected in pancreas. In diabetic mice, there is a coupled (serum and pulmonary) alteration in ACE activity that suggests that pulmonary and circulating RAS may exert a common regulation. The increase of ACE2 activity within the circulation in diabetic mice may be related to a compensatory RAS mechanism.

## 4. Materials and Methods

### 4.1. Animal Models

NOD/ShiLtJ and NOR/LtJ female mice (from The Jackson Laboratory, Bar Harbor, ME, USA) were housed in cages under 12 h light/dark cycle in a specific pathogen germ free (SPF) environment. Female mice only were used because the development of diabetes is more predictable in female than in male NOD mice [43]. Mice were fed with a chow diet and were provided access to tap water ad libitum. The Ethical Committee of Animal Experimentation of the Barcelona Biomedical Research Park (CEEA-PRBB) (MSO-08-1106) and the Catalan Government (DMAH: 4097) approved this study. Mice had their blood glucose levels determined every two weeks starting at 10 weeks of age. Fasting blood samples from tail vein were obtained and used for glucose level determination with the ACCU-CHEK Compact^®^ (Roche, St. Cugat, Spain). Female NOD mice were considered diabetic when glucose blood level higher than 250 mg/dL was first detected. NOD diabetic mice were randomly assigned to two groups, without (DB) or with insulin treatment (DB + INS). For blood glucose levels control, insulin pellets (~0.1 U/24 hr/pellet, LinBit, LinShin Canada Inc, Toronto, Canada) were subcutaneously implanted under anesthesia with ketamine and medetomidine. After surgery, atipamezol was injected to revert the effects of medetomidine. Diabetic animals were compared to the non-diabetic strain, NOR (CONT). Studied animals were weekly controlled for body weight and glucose blood levels and were followed for 21 and 40 days after diabetes diagnosis, and then final surgery was performed. The total number of animals included in each study group were the following: eight animals in each CONT group, seven animals in each DB group, and five animals were studied for 21 days with insulin pellet and six animals for 40 days with insulin treatment.

### 4.2. Mouse Tissue Samples

Studies were performed in serum, heart, lungs, liver, and pancreas. Animals were sacrificed under anesthesia, with pentobarbital. Blood samples were obtained by intracardiac puncture and organs were next perfused with Phosphate-buffered saline (PBS) solution by transcardiac puncture. Serum was obtained after 10 min of centrifugation at 6000× *g* and stored at −80 °C. Tissues were quickly removed and snap frozen in liquid nitrogen. They were then stored at −80 °C until use.

### 4.3. Determination of ACE Activity

ACE activity was first set up using human recombinant ACE (hrACE) and, for this reason, two different buffers were used with increasing concentrations of recombinant. One buffer contained 0.5 M potassium phosphate (PB) pH 8.3 [34] and the other was a mix of 0.4 M borate buffer (BB) pH 7.2, 0.34 M sucrose, and 0.9 M NaCl [32]. After that, increasing amounts of hrACE were incubated with 27.3 M Hip-His-Leu at 37 °C for 25 min. Reaction was stopped with 0.28 M NaOH and then 20 mg/mL of *o*-phtaldialdehyde in methanol were added to generate an adduct formation. The reaction was incubated at room temperature (RT) in dark conditions for 10 min and then it was stopped using 3 N HCl. Samples were clarified for 5 min at 800× *g* and His-Leu fluorescent adduct was measured fluorometrically at 360-nm excitation and 485-nm emission using a fluorescence plate reader Tecan Infinite 200 (TECAN Instruments, Männedorf, Switzerland). For mouse samples, BB was used following the protocol previously described. For serum, 2 μL of sample were used and for tissues, between 5 and 10 μg of total protein were analyzed depending on the tissue. Results were expressed as RFU (Relative Fluorescent Units) per μL of serum or μg of protein (RFU/μL or RFU/μg).

### 4.4. Determination of ACE2 Activity

ACE2 enzymatic activity assay was performed as previously published by our group [25,44,45,46] and adapted to different tissues. Briefly, 5 μL of serum or 5 μg of tissue samples that were previously homogenized were incubated using a 100 mM Tris-HCl, 600 mM NaCl, 10 μM ZnCl_2_, pH 7.5 buffer in the presence of protease inhibitors containing 100 μM captopril, 5 μM amastatin, 5 μM bestatin (all from Sigma-Aldrich, Madrid, Spain), and 10 μM Z-Pro-prolinal (Enzo Life Sciences, Grupo Taper, Madrid, Spain). Samples were incubated with 20 μM Mca-Ala-Pro-Lys(Dnp)-OH (Enzo Life Sciences), a specific ACE2 quenched fluorogenic substrate, at 37 °C. Enzymatic activity was determined after 4 hours of incubation in tissue, and 16 h of incubation in serum. The plates were read using a fluorescence plate reader Tecan Infinite 200 (TECAN Instruments) at an excitation wavelength of 320 nm and an emission wavelength of 400 nm. Results were expressed as RFU (Relative Fluorescent Units) per μL of sample or μg of protein and per hour (RFU/μl/h or RFU/μg/h).

### 4.5. Immunoblotting

Protein expression was analyzed by Western Blotting techniques using tissue homogenates. Briefly, 30 μg of protein were denatured by heat shock. Samples were loaded into 8% acrylamide/bisacrylamide gel and transferred to hydrophobic PVDF (polyvinylidenedifluoride) membranes (Amersham Hybond-P, GE Healthcare, Madrid, Spain) using Trans-Blot^®^ Turbo™ Transfer System (Bio-Rad Laboratories, Madrid, Spain). Membranes were blocked using 5% skimmed milk in Tris-buffered saline (TBS) containing 0.1% Tween-20 for 1 h at room temperature. Membranes were then incubated using primary antibodies for ACE (F940 1:500, Bioworld, St. Louis Park, MN, USA) and ACE2 (Ab15347 1:2000, Abcam, Cambridge, UK), followed by incubation with HRP-conjugated secondary antibodies (Dako, Barcelona, Spain). To control for protein loading, all membranes were probed with mouse monoclonal anti-β-actin (A1978 1:4000, Sigma, Madrid, Spain) or mouse monoclonal anti-tubulin (T5168 1:10000, Sigma). Densitometric analyses of protein bands were performed using ImageJ software (1.47v, NIH, USA) and corrected by control protein.

### 4.6. Statistical Analysis

Values of each data are expressed as mean ± SEM. Comparisons between groups were assessed by Kruskal-Wallis for multiple comparisons and Mann-Whitney *U*-test for two group comparisons (SPSS version 18 for Windows). Statistical significance was considered when *p* ≤ 0.05.

## Abbreviations

RAS	renin angiotensin system
AngII	angiotensin II
ACE	angiotensin converting enzyme
ACE2	angiotensin converting enzyme 2
NOD	non-obese diabetic
NOR	non-obese resistant
